# Supplementary material for: The interactome of intact mitochondria by cross-linking mass spectrometry provides evidence for coexisting respiratory supercomplexes
Source: Mol Cell Proteomics. 2017 Dec 8;17(2):216–32. doi: 10.1074/mcp.RA117.000470 (PMC5795388; doi:10.1074/mcp.RA117.000470)
Supplement: Supplemental Data [file supp_RA117.000470_133922_0_supp_23978_fzffwf.zip › spectra_annotation/mito_spectra_annotation/62-1-10-1-10-1.pdf]

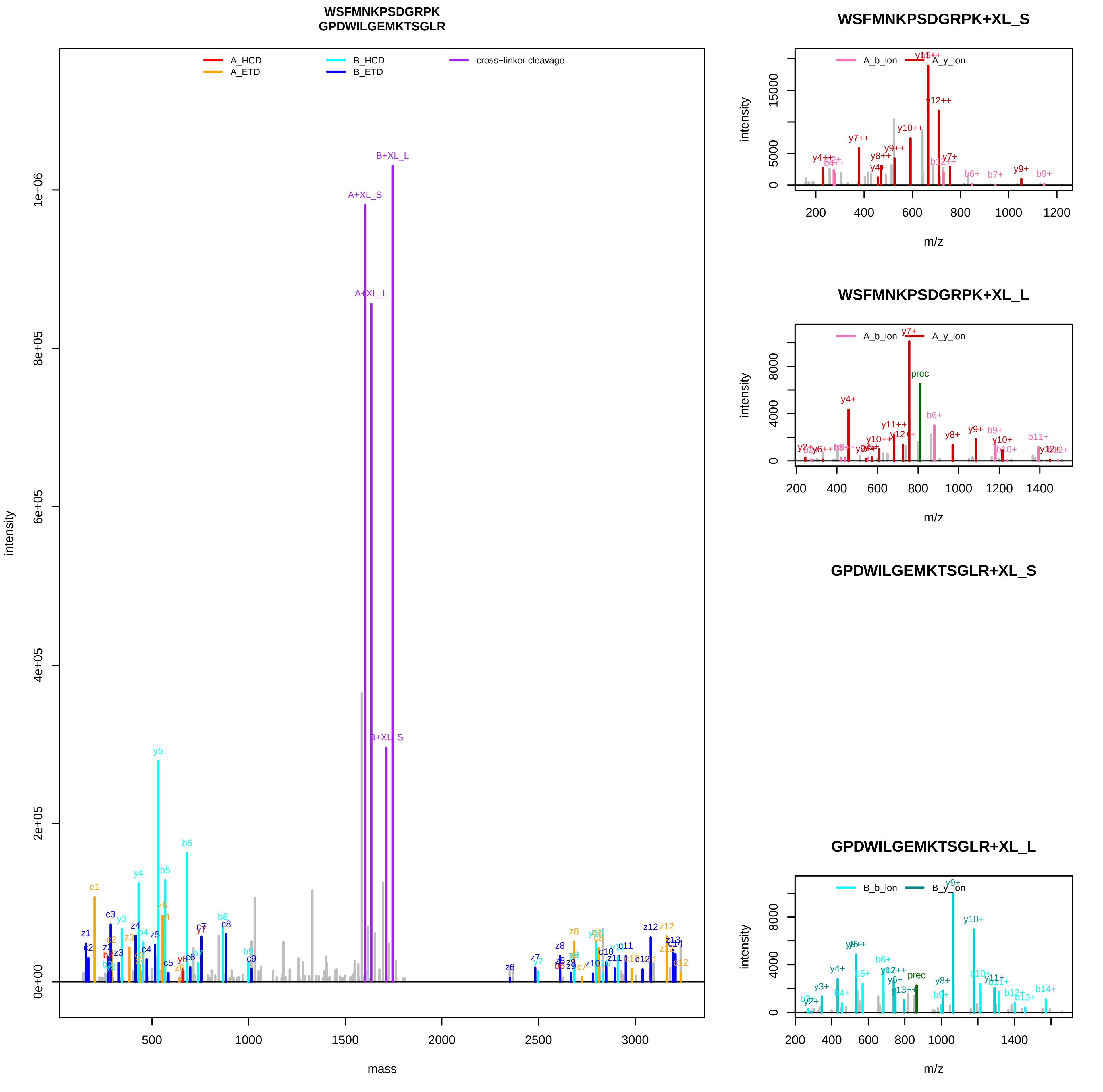
WSFMNKPSDGRPK
GPDWILGEMKTSGLR
A_HCD
A_ETD
B_HCD
B_ETD
cross−linker cleavage
B+XL_L
A+XL_S
A+XL_L
B+XL_S
intensity
mass
0e+00
2e+05
4e+05
6e+05
8e+05
1e+06
500
1000
1500
2000
2500
3000
WSFMNKPSDGRPK+XL_S
A_b_ion
A_y_ion
y11++
y12++
y10++
y7++
y9++
y8++
y4++
y7+
y9+
b6+
b7+
b9+
WSFMNKPSDGRPK+XL_L
y7+
prec
y4+
b6+
y11++
y12++
y8+
y9+
y10+
b9+
b11+
b10+
GPDWILGEMKTSGLR+XL_S
GPDWILGEMKTSGLR+XL_L
B_b_ion
B_y_ion
y9+
y10+
b6+
y4+
y3+
y11+
b14+
b13+
m/z
intensity
0
5000
15000
4000
8000
200
400
600
800
1000
1200
1400
